# Sex-associated TSLP-induced immune alterations following early-life RSV infection leads to enhanced allergic disease

**DOI:** 10.1038/s41385-019-0171-3

**Published:** 2019-05-11

**Authors:** Carrie-Anne Malinczak, Wendy Fonseca, Andrew J. Rasky, Catherine Ptaschinski, Susan Morris, Steven F. Ziegler, Nicholas W. Lukacs

**Affiliations:** 1 Department of Pathology, University of Michigan, Ann Arbor, MI USA; 2 Mary H Weiser Food Allergy Center, Ann Arbor, MI USA; 3 Department of Immunology, Benaroya Research Institute, Seattle, WA USA

## Abstract

Many studies have linked severe RSV infection during early-life with an enhanced likelihood of developing childhood asthma, showing a greater susceptibility in boys. Our studies show that early-life RSV infection leads to differential long-term effects based upon the sex of the neonate; leaving male mice prone to exacerbation upon secondary allergen exposure while overall protecting female mice. During initial viral infection, we observed better viral control in the female mice with correlative expression of interferon-β that was not observed in male mice. Additionally, we observed persistent immune alterations in male mice at 4 weeks post infection. These alterations include Th2 and Th17-skewing, innate cytokine expression (*Tslp* and *Il33*), and infiltration of innate immune cells (DC and ILC2). Upon exposure to allergen, beginning at 4 weeks following early-life RSV-infection, male mice show severe allergic exacerbation while female mice appear to be protected. Due to persistent expression of TSLP following early-life RSV infection in male mice, genetically modified TSLPR−/− mice were evaluated and demonstrated an abrogation of allergen exacerbation in male mice. These data indicate that TSLP is involved in the altered immune environment following neonatal RSV-infection that leads to more severe responses in males during allergy exposure, later in life. Thus, TSLP may be a clinically relevant therapeutic target early in life.

## Introduction

Respiratory syncytial virus (RSV) is often the first clinically relevant pathogen encountered, with nearly all children infected during the first 2 years of life.^1,2^ This viral infection is the leading cause of childhood hospitalization and increases the risk for developing childhood asthma and recurrent wheezing.^1,3^ The health burden costs of RSV infection account for over 3 million hospitalizations and ~100,000 deaths per year in children under 5, worldwide.^4,5^ Recent pre-clinical and clinical data, including data from our laboratory, suggest that severe RSV disease is associated with an altered innate and adaptive immune response, characterized by excessive Th2 and Th17 immune responses.^4,6–11^ Numerous studies have shown that RSV plays a role in reducing innate cytokine production that is necessary for appropriate anti-viral responses.^4,10,12,13^ Both viral replication as well as immunopathology can lead to RSV disease symptoms and probing these and potentially other underlying disease mechanisms that are supported by clinical data will be important for therapeutically targeting the immune environment.

During a viral infection, the immune response is strongly dictated by dendritic cells (DC) because they activate the immune system and instruct T cells toward distinct T helper type responses.^14^ RSV can skew the immune response away from anti-viral and towards a Th2-type response by inhibiting the production of IFN-β and subsequently decreasing the Th1 pro-inflammatory response.^15^ This lack of an anti-viral response as well as skewing towards dysregulated Th2/Th17 has been correlated with severe disease,^4,6,10^ leading to airway restructuring linked to exacerbated allergic responses later in life.^16^ Up to 48% of infants who were hospitalized for severe RSV-associated bronchiolitis and/or lower respiratory tract infection go on to develop asthma during their childhood.^17,18^ Asthma is the most common chronic childhood illness, affecting ~5 million children under 18 years of age, including 1.3 million children under 5.^19^ During childhood, males have higher occurrences of asthma and wheezing than do females at a 2:1 ratio.^20^ Additionally, males are approximately twice as likely to become hospitalized than females due to severe RSV infection.^21^ Thus, the risk for severe RSV infection and RSV-associated asthma development is higher in males than in females.^22^

How RSV alters the immune system to influence these observed responses is currently not well defined. Previous studies with neonatal RSV infection have demonstrated that there are persistent changes in the lung that include increased mucus production and increased populations of immune cells. Specifically, type 2 innate lymphoid cells (ILC2) are increased in RSV infected lungs and these cells produce IL-5 and IL-13, cytokines important in the development of inflammation and mucus production.^23,24^ Innate cytokines, including thymic stromal lymphopoietin (TSLP), IL-25 and IL-33 are known inducers of ILC2 differentiation.^25–27^ TSLP is a known driver of Th2-type responses through its influence on DCs, T cells, and ILC2s^28–30^ and may be required for CD4+ Th2 memory.^29^ RSV infection has been shown to lead to increased expression of TSLP,^31,32^ which has also been implicated in asthma pathogenesis.^33–35^ In fact, TSLP has been suggested as a possible biomarker in pediatric asthmatics.^36^ Additionally, it has been directly shown that RSV infection leads to activated IL-13 producing ILC2s that are dependent on TSLP.^37^ However, the mechanisms by which early life RSV infection alters immune responses related to allergen sensitization have yet to be elucidated.

In the present study, we examined the modulated immune responses due to neonatal RSV infection that include alteration of immune cell phenotypes and subsequent persistent changes leading to the altered development of allergic responses. Specifically, we demonstrate for the first time that early-life RSV infection alters immune cell populations in neonates in a sex-dependent manner through TSLP production to affect the early development of type 2 immune responses resulting in enhanced allergic responses later in life. These results suggest that clinically targeting TSLP during early-life RSV exposure may prevent RSV-induced immune environment changes and thus may decrease the incidence of childhood asthma.

## Results

### Neonatal RSV infection of male and female mice leads to a strong Th2/Th17-type immune response with delayed resolution in male mice

**Fig. 1:**
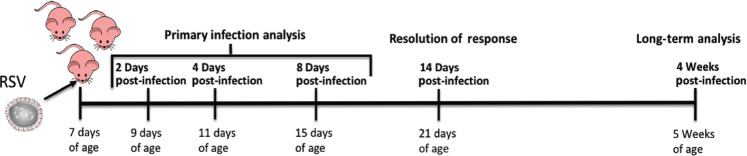
Neonatal RSV-infection experimental model

**Fig. 2:**
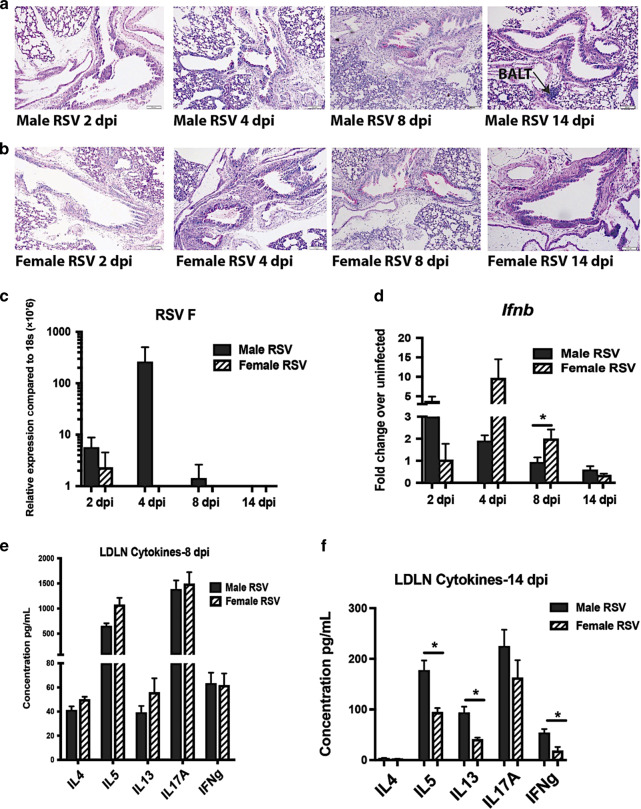
Neonatal RSV infection of male and female mice leads to a strong Th2/Th17-type immune response with delayed resolution in male mice. Male and Female mice were infected with RSV at 7 days of age and tissues collected at 2, 4, 8, or 14 days post-infection to evaluate primary response and resolution of Th2/Th17 response. **a**, **b** Lungs were embedded in paraffin and Periodic acid-Schiff stain (PAS) was performed to visualize mucus (bright pink staining) representative photos shown. **c**, **d** Lungs were homogenized and mRNA extracted to determine RSV F and interferon-β gene expression, respectively (*N* ≥ 3). **e**, **f** Lung draining lymph nodes were processed into single-cell suspension and re-stimulated with RSV in vitro for 48 h to determine cytokine protein levels (*N* ≥ 4). Data represent mean ± SEM (representative of at least 2 individual experiments). *p < 0.05

To evaluate the immune response to early-life RSV infection, male and female neonatal mice were infected intranasally with RSV (A2/L19-F (1.5 × 10^5^ pfu)) at 7 days of age (Fig. 1). Following infection, both males and females had mucus and inflammation present within their lungs peaking around 4–8 days post-infection with decreased mucus observed by 14 days post-infection (Fig. 2a, b). Age-matched male and female control mice show little to no signs of mucus production within the lungs at these time points (Supplemental Fig. 1), indicating viral induction of mucus. Additionally, at 14 days post-infection, lymphoid aggregates or bronchus associated lymphoid tissue (BALT) was observed in male mice, but not female mice (Fig. 2a). An increase in viral gene expression was observed in male compared to female mice on day 4 of infection (Fig. 2c) with female but not male mice having an increase in interferon-β gene expression (Fig. 2d), a critical cytokine required for viral clearance.^38^ Lung draining lymph nodes, harvested from neonatally infected mice at 8 days post RSV-infection, showed a strong Th2 and Th17-type immune response, with no discernible difference between males and females (Fig. 2e). However, upon analysis at 14 days post-infection, males showed higher cytokine responses in restimulated lymph nodes compared to females (Fig. 2f). These data indicate that both male and female neonatal mice elicit an RSV-driven Th2/Th17-type immune response but male mice displayed a lack of viral control with a delay in immune contraction compared to female mice.

### Male mice infected with RSV during early-life show signs of lung immunopathology that persists for up to 4 weeks post-infection

**Fig. 3:**
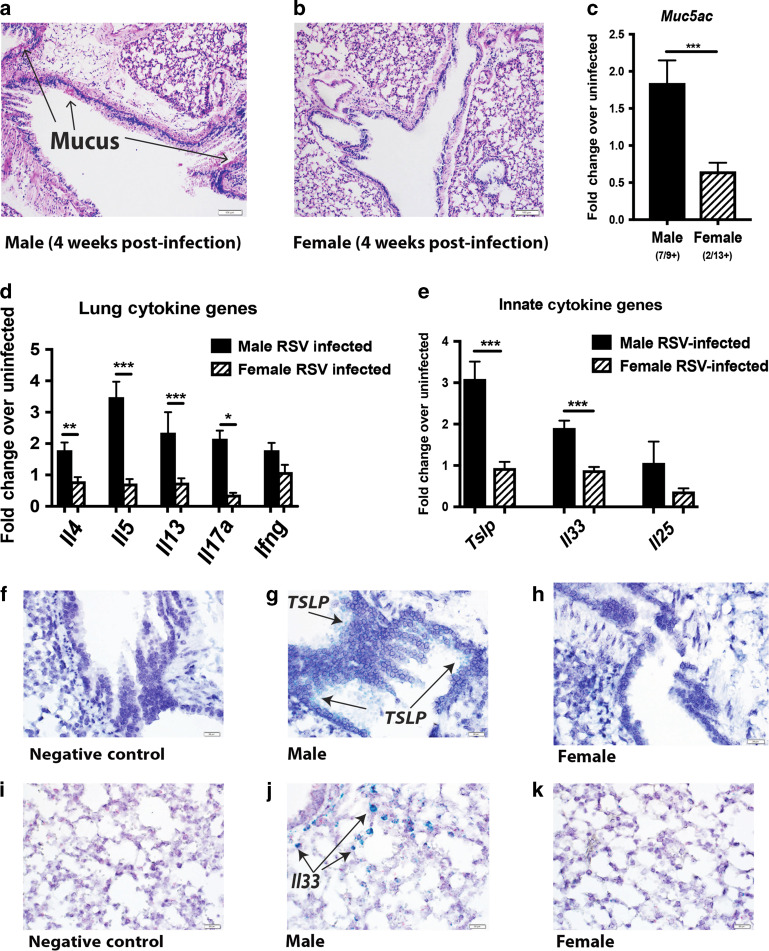
Male mice infected with RSV during early-life show signs of lung immunopathology that persists for up to 4 weeks post-infection. Male and Female mice were infected with RSV at 7 days of age and tissues collected at 4 weeks post-infection to evaluate long-term alterations. **a**, **b** Lungs were embedded in paraffin and Periodic acid-Schiff stain (PAS) was performed to visualize mucus (bright pink staining). Representative photos shown **c**–**e**. Lungs were homogenized and mRNA extracted to determine gene expression compared to age/sex-matched uninfected controls (*N* = ≥ 9) **f**–**k**. RNAscope was performed to determine location of *Tslp* and *Il33* within male lungs (stained in green). Representative photos shown. Data represent mean ± SEM (representative of or pooled from 2 individual experiments). *p < 0.05; **p < 0.01; ***p < 0.001

Further evaluation of persistent immune alterations was performed at later time points after neonatal RSV infection in male and female mice (Fig. 1). Uninfected, age-matched (5-week-old) mice were used as controls and no differences were observed between the sexes (Supplementary Fig. 2). However, significant differences were observed at 4 weeks post-early-life RSV-infection, with persistent lung immunopathology, including visible signs of mucus still present in the lungs of male mice (Fig. 3a) but not female mice (Fig. 3b). In correlation with lung pathology, males had increased expression of the mucus gene, *muc5ac*, compared to neonatally RSV-infected females (Fig. 3c). The number of mice that displayed visible mucus and overexpressed *muc5ac* compared to naïve at 4 weeks post-infection was 78% of early-life infected male mice (7/9) and 15% of female mice (2/13) (Fig. 3c). In addition, the results showed persistent gene expression of Th2-type and *Il17a* in males compared to females (Fig. 3d). Innate cytokines TSLP, IL-25, and IL-33, which are all strong drivers of Th2-type responses^39–41^ were also evaluated and indicated that early life RSV infected male mice showed persistent gene expression of *Tslp* and *Il33* compared to female mice (Fig. 3e). To determine the location of the *Tslp* and *Il33* within the male lungs, as these cytokines are strongly linked to asthma pathogenesis, studies were performed using RNAscope technology. *Tslp* expression was observed within the bronchial airway epithelial cells, while *Il33* was limited to the alveolar epithelial cell areas (Fig. 3f–k). Thus, early-life RSV infection in male mice leads to persistent Th2/Th17 skewing within the local lung environment, while females resolve this response and have a local lung environment similar to previously uninfected mice.

### Differential immune cell populations in the lungs of male and female mice with early-life RSV infection

**Fig. 4:**
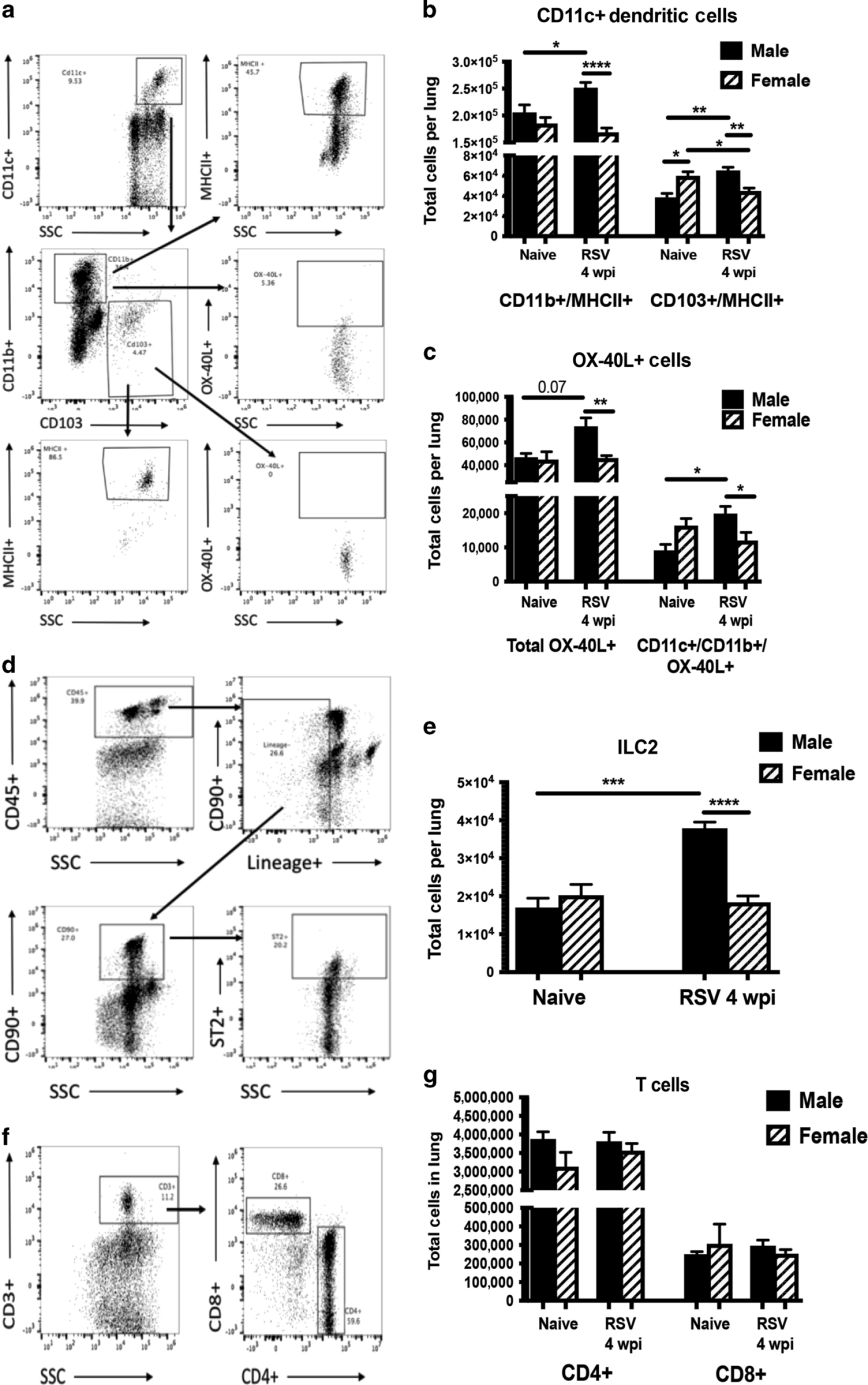
Differential immune cell populations in the lungs of male and female mice with early-life RSV infection. Male and Female mice were infected with RSV at 7 days of age and lungs collected at 4 weeks post-infection to evaluate cell populations. Lungs were processed into single-cell suspension and stained for flow cytometry analysis. **a** Gating strategy for dendritic cells. **b** CD11c+ dendritic cell types (*N* ≥ 9). **c** OX-40L+ cells (*N* ≥ 9). **d** Gating strategy for innate lymphoid cells. **e** Innate lymphoid cell populations (*N* = 5). **f** Gating strategy for T cells. **g** T cell populations (*N* ≥ 9). Data represent mean ± SEM (representative of or pooled from 2 individual experiments). *p < 0.05; **p < 0.01; ***p < 0.001; ****p < 0.0001

Due to the persistent immunopathology observed in the lungs from early-life RSV-infected male mice, we next examined immune cell populations that may lead to these alterations at 4 weeks post-infection. Age-matched (5-week old) naïve animals were used as controls. Naïve male and female animals showed similar immune cell populations at 5 weeks of age, indicating no inherent differences between the sexes. In contrast, lungs from male mice at 4 weeks post-neonatal RSV infection contained increased Cd11c+/Cd11b+ and Cd103+ DC populations (Fig. 4a, b). Interestingly, female mice at 4 weeks post-neonatal RSV infection also have decreased Cd103+ DC compared to naïve female mice (Fig. 4b). Additionally, early-life RSV infected males contain significantly more OX-40L+ cells than early-life infected females (Fig. 4a, c), which have been implicated in severe neonatal RSV responses as well as asthma.^31,42,43^ We also observed that males had significantly more ILC2, which can produce Th2-type cytokines (Fig. 4d, e). However, no differences were observed in CD4+ or CD8+ T cells (Fig. 4f, g). This phenotypic immune alteration in males may allow for the continued production of potentially pathogenic cytokines within the local environment.

### Early-life RSV infection leads to exacerbated allergic response in male mice

**Fig. 5:**
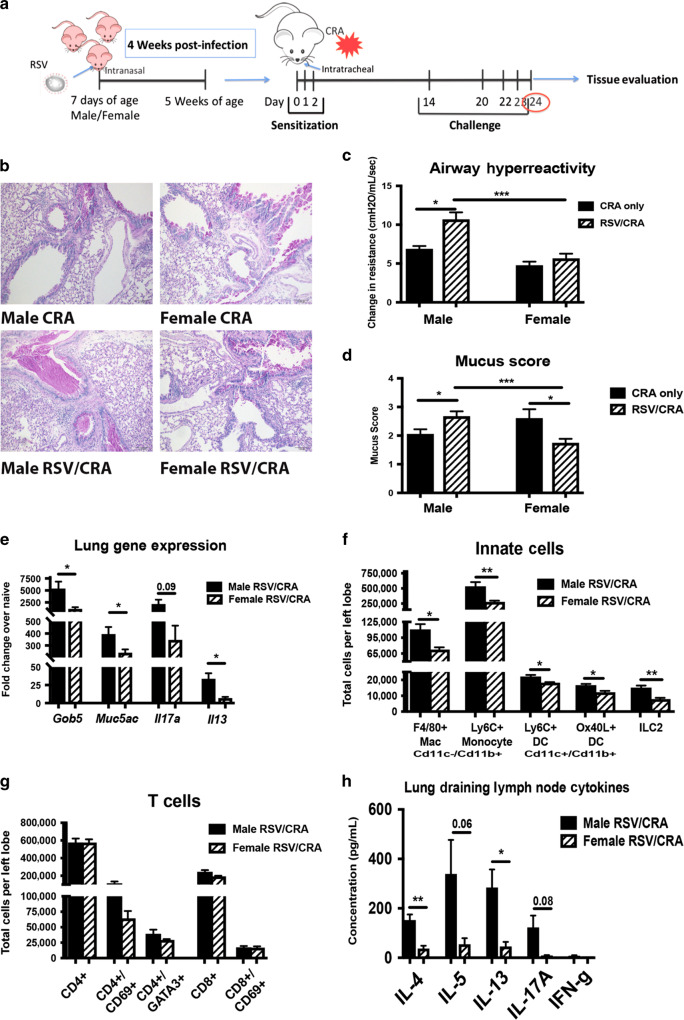
Early-life RSV infection leads to exacerbated allergic response in male mice. Male and Female mice were infected with RSV at 7 days of age and secondary allergen challenge initiated at 4 weeks post-infection. **a** Experimental Design. **b** Lungs were embedded in paraffin and Periodic acid-Schiff stain (PAS) was performed to visualize mucus (bright pink staining). Representative photos shown **c**. AHR was determined using full-body plethysmography and methacholine challenge (*N* ≥ 3). **d** Subjective mucus scoring was performed on blinded histological slides on a scale of 1–4 for mucus production (*N* ≥ 8). **e** Lungs were homogenized and mRNA extracted to determine mucus and cytokine gene expression compared to age/sex-matched naïve controls (*N* ≥ 3). **f**, **g** Lungs were processed into single-cell suspension and stained for flow cytometry analysis. (*N* ≥ 4). **h** Lung draining lymph nodes were processed into single cell suspension and re-stimulated with CRA in vitro for 48 h to determine cytokine protein levels (*N* ≥ 3). Data represent mean ± SEM (representative of or pooled from 2 to 3 individual experiments). *p < 0.05; **p < 0.01; ***p < 0.001

Five-week-old mice that were previously infected with RSV at 7 days of age were sensitized and challenged with cockroach allergen (CRA) to determine if prior infection with RSV alters the immune response to allergens later in life (RSV/CRA). CRA was administered via intratracheal instillation into the lungs over 3 consecutive days, starting at 4 weeks post-infection, followed by 4 challenges 2 weeks later to elicit an allergic response (Fig. 5a). Naïve age-matched animals were used as controls and administered CRA in the same manner. Lung histology showed that RSV/CRA males have severe pathology and increased mucus compared both to mice given only CRA and female mice given RSV/CRA (Fig. 5b). Airway hyperreactivity (AHR) was significantly higher in the males given RSV/CRA (Fig. 5c) compared to all other groups indicating more severe lung physiology responses. A correlative increase in mucus score was also observed in the RSV/CRA male mice (Fig. 5d) and interestingly, a decreased mucus score was observed in RSV/CRA female mice compared to both RSV/CRA male mice as well as CRA only female mice (Fig. 5d). Additionally, mucus-associated genes, *gob5* and *muc5ac*, as well as gene expression of *Il13* were significantly increased in lungs from male mice given RSV/CRA, compared to female mice (Fig. 5e). Increased infiltration of inflammatory myeloid-type cells as well as ILC2 were observed in male mice given RSV/CRA compared to females given RSV/CRA, with no differences in T cell numbers (Fig. 5f, g). However, analysis of the CRA-specific response analyzed by cytokine production from isolated lymph node cells showed that male mice infected with RSV during early life had significantly more Th2 and IL-17A cytokine production upon CRA re-challenge when compared to females, suggestive of a shift in T cell responsiveness to the allergen (Fig. 5h). No differences were observed between uninfected males and females tested (Supplemental Fig. 3), indicating that the baseline allergic response is equivalent between male and female mice. Altogether, these data indicate that the effects of early-life RSV-infection lead to sex-specific alterations that persist long-term in male mice.

### Deletion of TSLP receptor leads to decreased immune responses following CRA exposure in male mice with early-life RSV infection

**Fig. 6:**
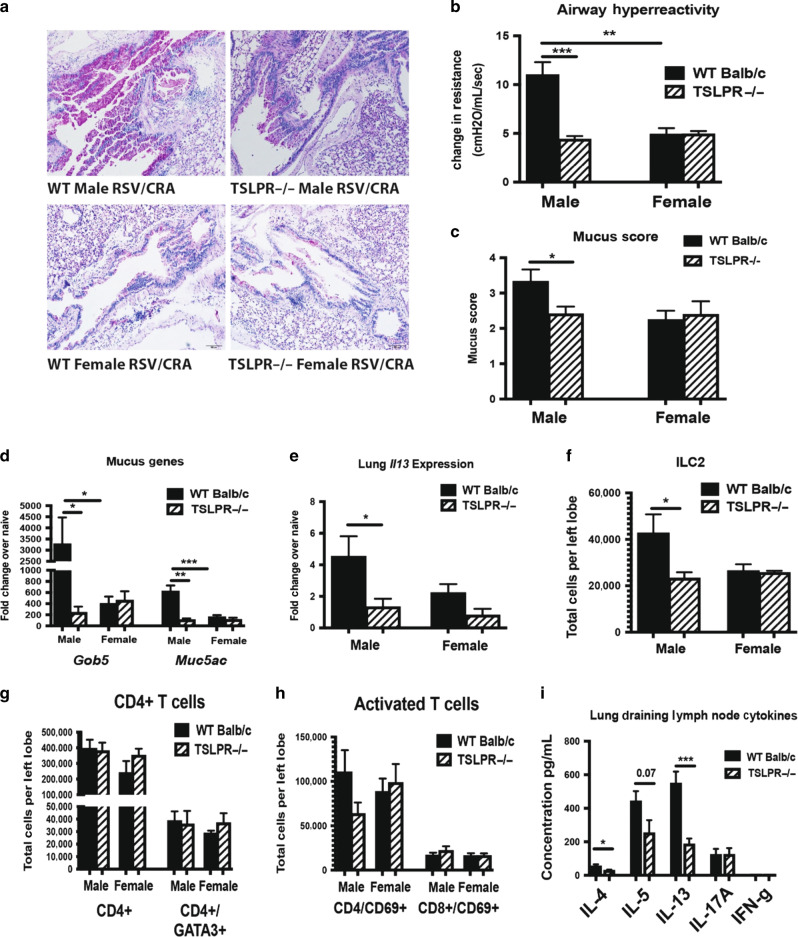
Deletion of TSLP receptor leads to decreased immune responses following CRA exposure in male mice with early-life RSV infection. Male and Female TSLPR−/− mice were infected with RSV at 7 days of age and secondary allergen challenge initiated at 4 weeks post-infection. **a** Lungs were embedded in paraffin and Periodic acid-Schiff stain (PAS) was performed to visualize mucus (bright pink staining). Representative photos shows **b** AHR was determined using full-body plethysmography and methacholine challenge (*N* ≥ 3). **c** Subjective mucus scoring was performed on blinded histological slides on a scale of 1–4 for mucus production (*N* ≥ 6). **d**, **e** Lungs were homogenized and mRNA extracted to determine mucus and cytokine gene expression compared to age/sex-matched naïve controls (*N* ≥ 3). **f**–**h** Lungs were processed into single-cell suspension and stained for flow cytometry analysis (*N* ≥ 3). **i** Lung draining lymph nodes in single-cell suspension were re-stimulated with CRA in vitro for 48 h to determine cytokine protein levels (*N* ≥ 3). Data represent mean ± SEM (representative of or pooled from 2 to 3 individual experiments). *p < 0.05; **p < 0.01; ***p < 0.001

Based on our observed correlative evidence of TSLP expression with persistent Th2/Th17-skewing, we evaluated the sex-associated role of TSLP in the immune exacerbation observed following CRA exposure in mice. Male and female TSLP receptor knockout (TSLPR−/−) mice were RSV infected during early-life, followed by a CRA challenge initiated 4 weeks later (Fig. 5a). Histologic evaluation showed decreased mucus in the lungs of TSLPR−/− male mice compared to WT male mice given early RSV followed by the CRA challenge (Fig. 6a). Furthermore, AHR was decreased in TSLPR−/− male mice with a previous neonatal RSV infection (Fig. 6b), indicating improved lung function compared to WT male mice. Consistent with histology, a decrease in mucus score (Fig. 6c) as well as mucus gene expression (Fig. 6d) was observed in TSLPR−/− male mice compared to WT male mice given RSV/CRA. The results also demonstrated a decrease in *Il13* cytokine gene expression levels within the lungs of TSLPR−/− compared to WT male mice given RSV/CRA (Fig. 6e). Correlating with the decreased *Il13* expression, a decrease in ILC2 was observed in the TSLPR−/− male mice given RSV/CRA (Fig. 6f) with no changes in CD4+ T cell numbers (Fig. 6g, h). Additionally, male TSLPR−/− RSV-infected/CRA challenged mice had decreased CRA-specific Th2 cytokine production in the lung draining lymph nodes compared to WT mice (Fig. 6i). Of interest, similar to previous studies,^44^ we found no difference in the male or female mice given only CRA when TSLPR−/− mice were compared to WT mice (Supplemental Fig. 3), suggesting that the TSLP-induced environment created by an early life RSV infection was critical. Importantly, unlike male mice, females were not exacerbated following RSV-infection alone and thus were independent of TSLP, supporting a sex-specific role for this pathway.

## Discussion

Clinically, data suggest that severe RSV infection during early-life enhances the likelihood of developing childhood asthma by 3–5 fold.^1,45^ Most children are infected with RSV within the first 2 years of life and up to 3–5% of children become hospitalized due to complications from severe disease.^21^ Studies suggest that ~50% of children hospitalized during severe RSV infection go on to develop childhood asthma.^17,18^ Additionally, clinical data show that boys are more susceptible to RSV, hospitalized at a 2:1 ratio compared to girls, and are more than twice as likely to develop childhood asthma.^2,21,46^ Here we show that male mice infected neonatally with RSV have poorer viral control than female mice of the same age and that at 4 weeks post-early-life RSV-infection, male mice show a persistent signature of RSV-driven Th2/Th17 immunopathology, mucus and an altered immune environment that leads to an exacerbated allergic phenotype associated with TSLP driven mechanisms. Thus, these studies outline 2 important concepts; (1) early RSV infection results in an altered immune phenotype in the lung that is dependent upon sex; and (2) the persistence of the early innate cytokine, TSLP, has a role in the altered immune environment and may be a clinically relevant target early in life.

TSLP is an early responding, innate cytokine, that can drive Th2-type responses and is upregulated following RSV infection of lung epithelial cells.^32^ During neonatal RSV-infection, TSLP mediates immunopathology through OX-40/Th2 signaling,^31^ as well as having direct effects upon ILC2 cells, causing them to produce Th2-type cytokines, IL-5 and IL-13 to enhance inflammation and mucus production.^37^ When TSLP was neutralized during neonatal RSV infection, the severity of a later exacerbated RSV infection was mitigated,^31^ indicating that TSLP may be creating a broadly altered immune environment. Additionally, TSLP has been implicated in the pathogenesis of asthma. Increased TSLP mRNA in bronchial epithelial cells of asthmatics directly correlates with degree of airflow obstruction^47^ and GWAS studies show a strong association between TSLP and severe asthma.^48,49^ The TSLP SNP, rs1837253 on the T allele, which leads to decreased epithelial cell production of TSLP, has been identified in boys to reduce the risk of developing atopic asthma and airway hyperresponsiveness as well as protection from allergic rhinitis.^50–52^ This same TSLP mutation shows no protective effect in girls, suggesting that the role of TSLP in asthma may be more significant in boys than girls. Our data expands on these clinical findings, showing a significant correlation between RSV-induced TSLP and the subsequent development of viral-associated allergic asthma later in life in male mice, following early-life RSV-infection.

TSLP has a wide range of immune cell targets, including ILC2, DC, and T cells. The initial biologic function of TSLP demonstrated that it interacted with DC to promote Th2 cell differentiation and thus promoted allergic disease.^41,53^ TSLP stimulation of CD11b+ DC upregulates OX-40L that interacts with OX-40 on CD4 T cells to promote a Th2 response.^42,43^ The survival and maintenance of ILC2 that produce Th2-type cytokines, depends on the presence of TSLP.^54,55^ In addition, TSLP signaling instructs DCs to produce CCL-22,^28^ a chemokine that recruits Th2 cells through its interaction with the CCR4 receptor. Finally, in response to TSLP, DCs will themselves produce TSLP that further interacts with DCs and ILC2, creating a feedback loop that is independent of epithelial cell-derived TSLP.^56,57^ The data provided in our studies, using TSLPR−/− mice, indicated that TSLP signaling is responsible for the increased presence of innate immune cells in the lungs of male mice conditioned with a neonatal RSV infection. Interestingly, the TSLPR−/− mice had a similar allergen response in the lungs compared to WT male and female mice when no neonatal RSV infection was given. These data recapitulate previous data indicating that although TSLP is involved in the development of Th2 cells, the long-term allergen responses are not altered in its absence.^44^ Similarly, TSLP was not necessary to develop chronic Th2 driven parasitic helminth granulomatous responses.^58^ Thus, TSLP may be central to the development of chronic type 2 immune responses when a stimulus, such as RSV infection, promotes an altered immune environment that predisposes tissue to development of allergic responses. This is likely more significant in neonates that have an immature immune environment that can be more easily skewed to an altered immune phenotype.

The upregulation of another innate cytokine, IL-33, was also associated with the early RSV infection in male mice. While we have not further explored the role of IL-33 in the sex difference in the altered immune environment, it may also contribute. Interesting, the expression of TSLP mRNA was more highly evident and associated with bronchial epithelial cell area in male mice at 4-weeks post-infection, whereas IL-33 appeared to be more contained and associated with alveolar areas as assessed by RNAscope. Many studies have indicated a role for these innate cytokines in respiratory viral complications, asthma development, as well as viral exacerbations of existing asthma.^59–61^ It is evident that these cytokines have distinctive as well as additive effects on ILC2 responses.^27,54,62^ Allergen challenge, following neonatal rhinovirus infection identified that both IL-33 and TSLP are required for IL-25-induced ILC2 production of IL-13 leading to mucus metaplasia but TSLP was necessary for maximal ILC2 gene expression even in the presence of IL-25 and IL-33.^27^ Additionally, experimental asthma pathogenesis was shown to persist in IL-33R knockout mice (ST2−/−) due to increased expression of TSLP that enhanced ILC2 production of IL-13; abrogation of this response was only possible with the addition of an anti-TSLP antibody.^62^ Finally, in an allergen challenge model conducted on adult human subjects with allergic asthma, administration of an anti-TSLP monoclonal antibody (AMG 157) reduced allergen-induced bronchoconstriction and airway inflammation, suggesting a key role for TSLP in allergen-induced airway responses in asthmatics.^63^ Collectively, these studies as well as our data presented here make it plausible that TSLP, IL-25 and IL-33 cooperate and regulate each other during initial disease progression but TSLP may be more significant in the development of subsequent diseases later in life. Given the availability of anti-TSLP in human trials, a therapeutic intervention in infants with severe RSV may be highly beneficial, especially for boys.

These novel data recapitulate the clinical observations where males are more likely to develop severe respiratory complications and childhood asthma than are girls. This suggests that early-life RSV-infection in males leads to persistent TSLP-associated Th2/Th17 immune-driven pathology within the lungs, while the same early-life infection in females allows for appropriate Th2/Th17 resolution thereby protecting females from later asthma pathogenesis. This phenomenon may be due to poorly controlled viral replication and decreased viral clearance in males following the initial infection, possibly due to lack of type-1 interferon production, which is critical for viral clearance as well as resolution of Th2/Th17 immune responses.^38,64,65^ Correlating with our findings, it has previously been shown that female plasmacytoid dendritic cells (pDC) isolated from human peripheral mononuclear cells express higher levels of type-1 interferons (IFNα/β) compared to male pDC following TLR7 stimulation.^66^ Studies with bronchial epithelial cells from asthmatics have shown that TSLP and interferon-β have an inverse relationship, showing a bias towards low interferon-β but high TSLP, while the reverse is true for healthy individuals.^67^ A separate study has shown that type-3 interferon (IFNλ) has the ability to upregulate TSLP expression,^68^ the role of type-1 interferons and TSLP has not been fully elucidated. Overall, these results may suggest that targeting TSLP during early-life RSV infection, especially in male infants hospitalized with severe disease, may limit these changes and may decrease the incidence of childhood asthma.

## Materials and Methods

### Animals

All experiments involving the use of animals were approved by the University of Michigan animal care and use committee. Male and female BALB/c mice, 6 to 8 weeks of age, were purchased from The Jackson Laboratory (Bar Harbor, ME) and used as breeders for experimental animals. TSLPR−/− mice were kindly provided by Dr. Steven Ziegler and adult mice bred in-house were used as breeders. Male and female mice (WT and TSLPR−/−), born in-house were used for all experiments. All mice were maintained under standard pathogen-free conditions.

### RSV-infection

A chimeric RSV A2 strain with recombinant Line19 fusion protein was used for all experiments as previously described.^69^ Male and female Balb/c and TSLPR−/− mice were infected intranasally (5 µL/animal) with 1.5 × 10^5^ pfu of RSV A2/L19-F at 7 days of age.

Analysis of the viral response was performed at 2–8 days and 14 days post-infection to determine primary response and resolution, respectively. Long-term analysis was performed at 4 weeks post-infection (Fig. 1).

### Mouse CRA asthma model

The allergen used was a clinical grade, skin test CRA (Hollister-Stier, Spokane, WA) as previously described.^70,71^ Mice were sensitized intratracheally with 500 protein nitrogen units (pnu) of CRA, over 3 consecutive days. Next, mice were challenged intratracheally with 500 pnu of CRA on days 14, 20, 22, and 23 after initial CRA sensitization. On day 24, one day after the last allergen challenge, animals were killed, and samples were taken. The same model was used in the TSLPR−/− mice.

### Quantitative RT-PCR

Lung tissue was homogenized in TRIzol reagent and RNA was extracted using TRIzol reagent (Invitrogen, Carlsbad, CA). cDNA was synthesized using murine leukemia virus reverse transcriptase (Applied Biosystems, Foster City, CA) and incubated at 37 °C for 1 h, followed by incubation at 95 °C for 10 min to stop the reaction. Real-time quantitative PCR (qPCR) was multiplexed using Taqman primers, with a FAM-conjugated probe to measure transcription of *Gob5, Muc5ac, Il4, Il5, Il13, Il17a, Ifng, Tslp, Il25*, and *Il33*. Fold change was quantified using 2^−**ΔΔ**^ cycle threshold (CT) method. Custom primers were designed to measure Muc5ac and Gob5 mRNA levels as described.^72^ All reactions were run on a 7500 Real-Time PCR System (Applied Biosystems).

### Lung histology

The 2 middle lobes of the right lung were perfused with 10% formalin for fixation and embedded in paraffin. Five-micrometer lung sections were stained with periodic acid-Schiff (PAS) to detect mucus production, and inflammatory infiltrates. Photomicrographs were captured using a Zeiss Axio Imager Z1 and AxioVision 4.8 software (Zeiss, Munich, Germany).

### Flow cytometry

The lungs were removed, and single cells were isolated by enzymatic digestion with 2.5 mg/ml Liberase^TM^ (Roche) and 20 U/ml DNaseI (Sigma, St. Louis, MO) in RPMI 1640 for 45 min at 37 °C or 1 mg/mL collagenase (Roche) and 20 U/ml DNaseI (Sigma, St. Louis, MO) in RPMI 1640 + 10% FCS for 60 min at 37 °C. Tissues were further dispersed through an 18-gauge needle (5-ml syringe), RBCs were lysed and samples were filtered twice through 100-μm nylon mesh. Cells were resuspended in PBS. Live cells were identified using LIVE/DEAD Fixable Yellow Dead Cell Stain kit (Thermo Fisher Scientific, Waltham, MA), then washed and resuspended in PBS with 1% FCS. Fc receptors were blocked with purified anti-CD16/ 32 (clone 93; BioLegend, San Diego, CA). Surface markers were identified using Abs (clones) against the following antigens, all from BioLegend: anti-Cd11c (N418), MHC II (M5/114.15.2), Cd11b (M1/70), OX-40L (RM134L), Cd3 (145–2C11), Cd4 (GK1.5), Cd8 (53–6.7), Cd25 (3C7), Cd90 (53–2.1), cKit (2B8), ST2 (D1H9), Gr-1 (RB6- 8C5), B220 (RA3–6B2), Ter119 (Ter-119). For innate lymphoid cell staining, lineage markers were anti-CD3, CD11b, B220, Gr-1, and TER119. ILC2: CD45+ /Lin-/ CD90+ / ST2+. Data was collected using a NovoCyte flow cytometer (ACEA Bioscience, Inc. San Diego, California). Data analysis was performed using FlowJo software (Tree Star, Oregon, USA).

### Mediastinal lymph nodes in vitro restimulation and cytokine production assay

Mediastinal lymph nodes (mLN) were enzymatically digested using 1 mg/ml collagenase A (Roche) and 20 U/ml DNaseI (Sigma-Aldrich) in RPMI 1640 with 10% FCS for 45 min at 37 °C. Tissues were further dispersed through an 18-gauge needle (1-ml syringe). RBCs were lysed, and samples were filtered through 100-μm nylon mesh. Cells (5 × 10^5^) from mLN cells were plated in 96-well plates and restimulated with 1.5–3.0 × 10^5^ pfu of RSV or 300 pnu of CRA for 48 h. IL-4, IL-5, IL-13, Il-17a, and IFN-γ levels in supernatants were measured with a Bio-Plex cytokine assay (Bio-Rad Laboratories).

### RNAscope for formalin fixed paraffin embedded (FFPE) tissue

Lungs were perfused with 10% formalin for fixation and embedded in paraffin (FFPE). RNAscope 2.5 duplex detection kit for FFPE samples (ACDbio, Newark, CA) was used to perform RNAscope as previously described.^73^ Probes to detect *Tslp* (target region 10–1149) and *Il33* (target region 2–947) were used with green chromogen detection.

### Measurement of airway hyperreactivity

Airway hyperreactivity was measured using mouse plethysmography, that is specially designed for the low tidal volumes (Buxco Research Systems), as previously described.^70,74^ Briefly, the mouse to be tested is anesthetized with sodium pentobarbital and intubated via cannulation of the trachea with an 18-gauge metal tube. The intubated mouse was ventilated at a volume of 200 μL at a rate of 120 breaths/min. The airway resistance was measured in the closed plethysmograph by directly assessing tracheal pressure and comparing the level to corresponding box pressure changes. These values were monitored and immediately transformed into resistance measurements using computer-assisted calculations. Once baseline levels had stabilized and initial readings were taken, a methacholine challenge was given via iv tail vein injection (375 µg/kg of methacholine) as previously described.^70,74^ After the methacholine challenge, the response was monitored, and the peak airway resistance was recorded as a measure of airway hyperreactivity.

### Mucus scoring analysis

Slides from PAS-stained lungs were blind-coded and scored by an individual observer to quantify mucus on a scale of 1–4. Scoring is as follows: 1 = Minimal/No Mucus; 2 = Slight: Multiple airways with goblet cell hyperplasia and mucus; 3 = Moderate: Multiple airways with significant mucus and some plugging; 4 = Severe: significant Mucus plugging.

### Statistical analysis

Data were analyzed by Prism 7 (GraphPad Software). Data presented are mean values ± SEM. Comparison of two groups was performed with an unpaired, two-tailed Student’s *t*-test. Comparison of three or more groups was analyzed by one-way ANOVA, followed by two-tailed Student’s *t*-test for individual comparisons. A *p*-value < 0.05 was considered significant.

## Supplementary information

Supplementary Information
