# Supplementary material for: Sex-associated TSLP-induced immune alterations following early-life RSV infection leads to enhanced allergic disease
Source: Mucosal Immunol. 2019 May 11;12(4):969–79. doi: 10.1038/s41385-019-0171-3 (PMC6599479; doi:10.1038/s41385-019-0171-3)
Supplement: Supplementary file 1 — Supplementary Information [file 41385_2019_171_MOESM1_ESM.docx]

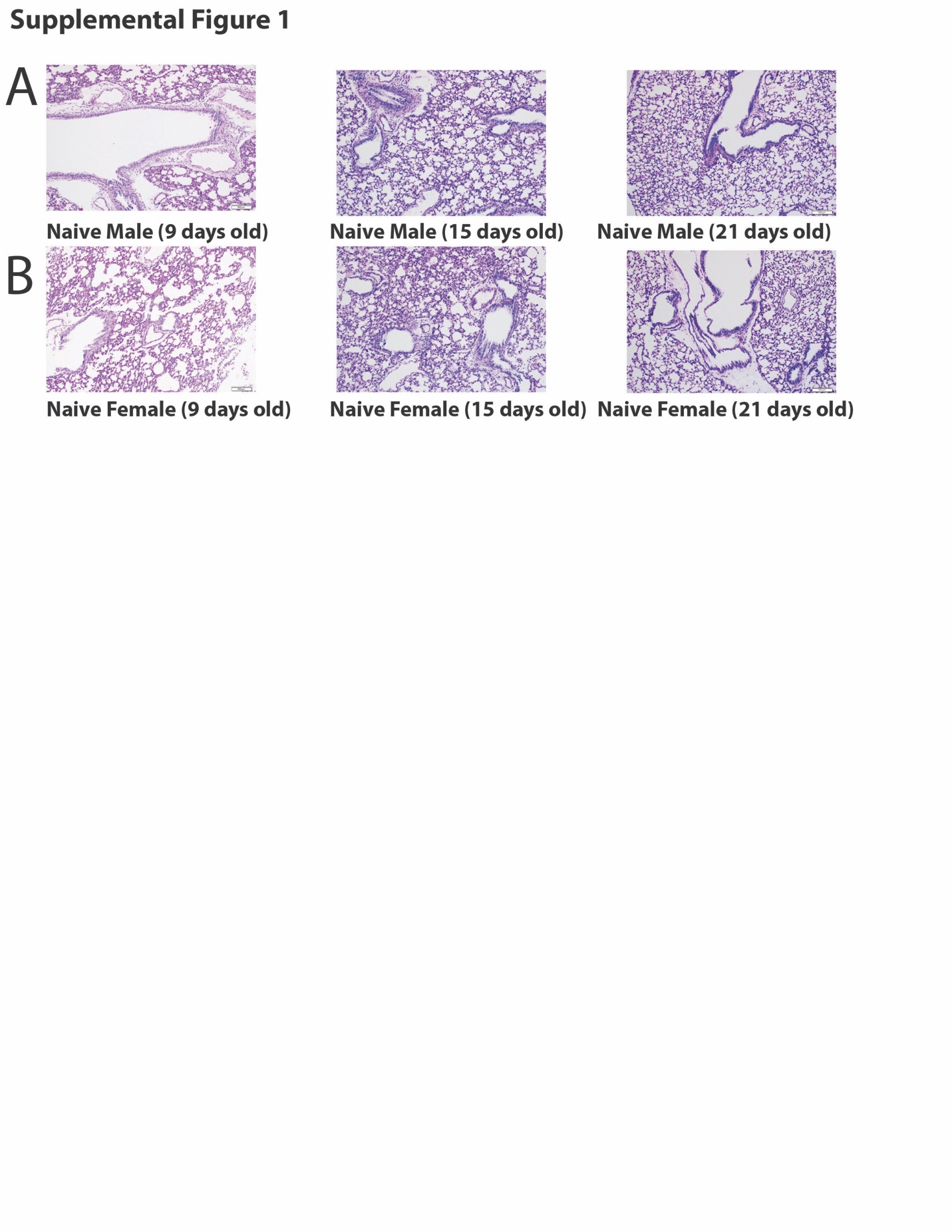


**Supplemental Figure 1**. **Naïve neonatal male and female mice have similar local lung environment.** Tissues were collected from age-matched male and female uninfected mice at the time of 2 (9 days old), 8 (15 days old), and 14 (21 days old) day post-infection analysis for infected mice. **A,B**. Lungs were embedded in paraffin and Periodic acid-Schiff stain (PAS) was performed. Representative photos shown


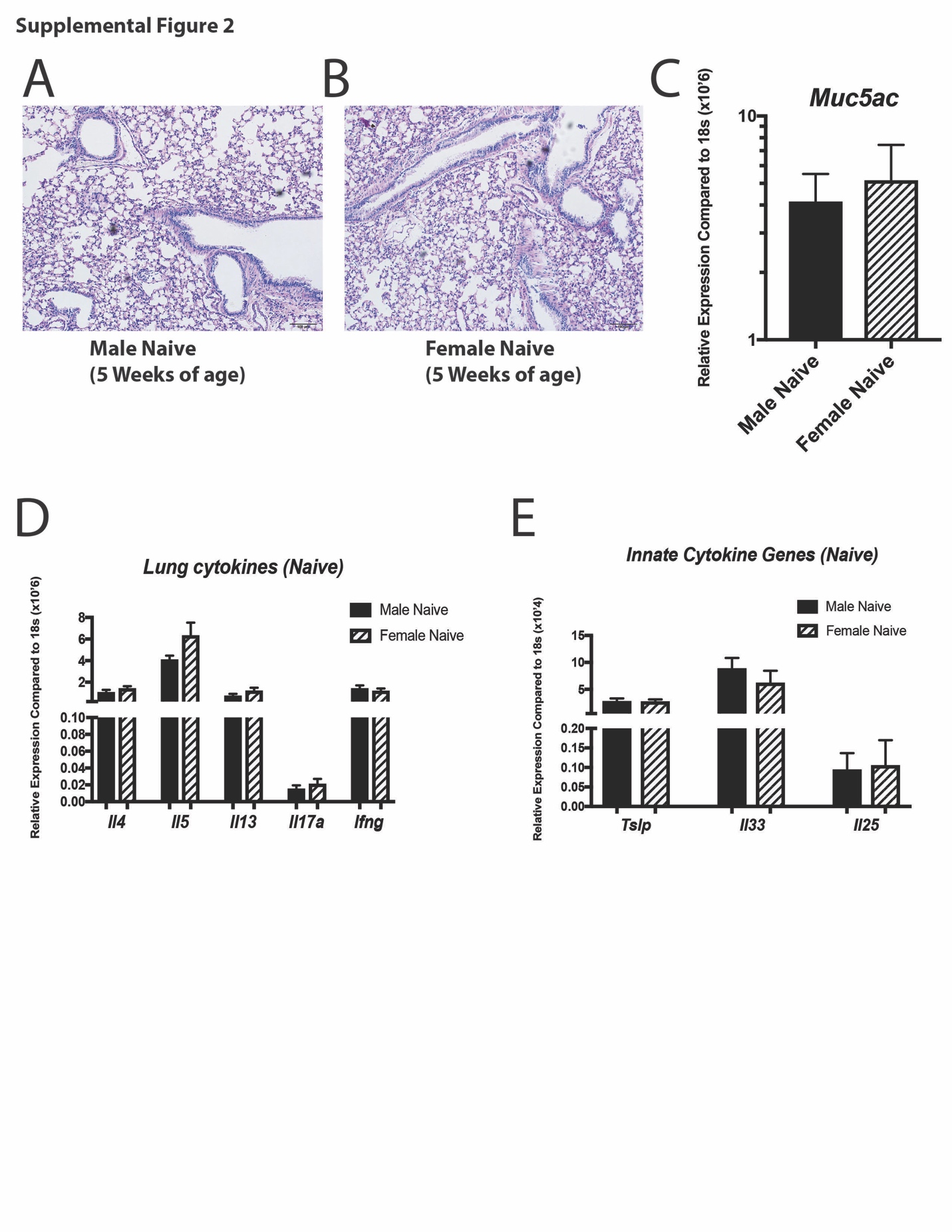


**Supplemental Figure 2**. **Naïve male and female mice have similar local lung environment at 5 weeks of age.** Tissues were collected from 5 week old male and female uninfected mice at the time of 4 week post-infection analysis for infected mice. **A,B**. Lungs were embedded in paraffin and Periodic acid-Schiff stain (PAS) was performed. Representative photos shown **C-E.** Lungs were homogenized and mRNA extracted to determine relative gene expression compared to 18s housekeeping gene (N = ≥ 3). Data represent Mean ± SEM (2 individual experiments)


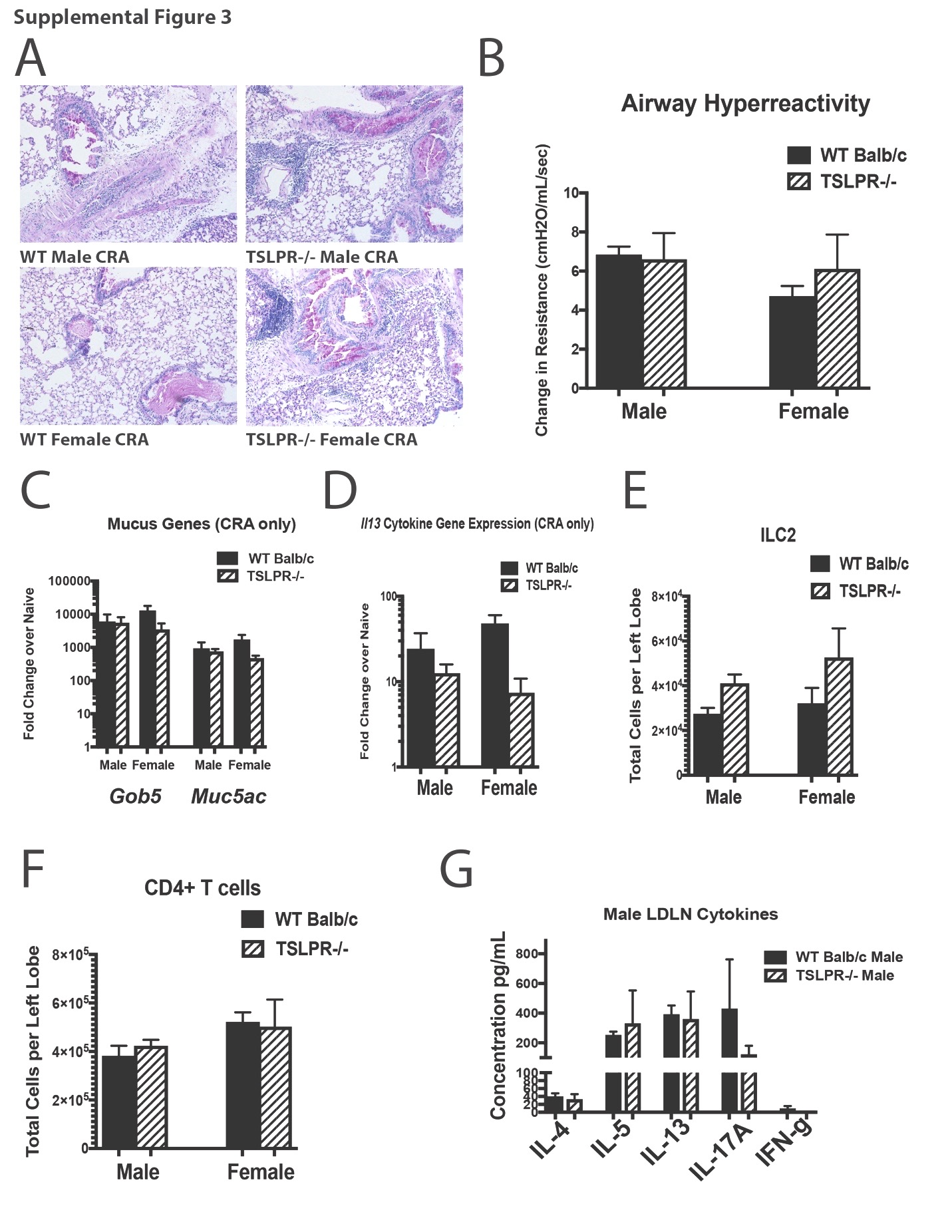


**Supplemental Figure 3. No significant differences are observed in male and female WT or TSLPR-/- mice following CRA challenge alone.** Male and Female WT and TSLPR-/- mice were exposed to CRA allergen challenge at 5 weeks of age (initiated at the time of 4 weeks post-infection for RSV/CRA animals). **A**. Lungs were embedded in paraffin and Periodic acid-Schiff stain (PAS) was performed to visualize mucus (bright pink staining). Representative photos shown **B**. AHR was determined using full-body plethysmography and methacholine challenge (N ≥ 3) **C, D**. Lungs were homogenized and mRNA extracted to determine mucus and cytokine gene expression (N ≥ 3) **E, F**. Lungs were processed into single-cell suspension and stained for flow cytometry analysis. (N ≥ 7) **G**. Lung draining lymph nodes in single cell suspension were re-stimulated with CRA *in vitro* for 48 hours to determine cytokine protein levels (N≥ 3). Data represent Mean ± SEM (2-3 individual experiments)
